# Allergy as an epithelial barrier disease

**DOI:** 10.1186/2045-7022-1-5

**Published:** 2011-06-10

**Authors:** Pirkko Mattila, Sakari Joenväärä, Jutta Renkonen, Sanna Toppila-Salmi, Risto Renkonen

**Affiliations:** 1 Transplantation Laboratory & Infection Biology Research Program, Haartman Institute, University of Helsinki & Helsinki University Central Hospital, HUSLAB, Helsinki, Finland

## Abstract

The objective of this review is to focus on putative modified epithelial functions related to allergy. The dysregulation of the epithelial barrier might result in the allergen uptake, which could be the primary defect in the pathogenesis of allergic reaction. We review the literature of the role of respiratory epithelium as an active barrier, how allergens are transported through it and how it senses the hostile environmental allergens and other dangerous stimuli.

## Introduction

Acute allergic reactions are known to be symptomatic type-I hypersensitivity responses caused by an allergen cross-linking specifically with anti-IgE molecules on the surface of pre-sensitized mast cells [1-4]. In Europe the incidence of allergies rapidly increasing and thus we are facing an epidemic outburst of these diseases [5]. Even though costs per a patient are usually small allergies cause a major economical burden to the society, since up to 20% of the population now suffers from these disorders for instance in Finland [6]. Because of this the Finnish authorities have launched an "Allergy project 2008-2018", where a new direction has been taken. Here the patients will now be tolerated towards the antigens instead of avoiding them [6].

Currently there are two, partially different basic theories on the pathogenesis of allergy. For decades the primary assumption has been that allergy is caused by unbalanced and overactive immunological responses against allergens, mostly driven by activated Th2 cells and due to aberrant T- regulatory cells. The second more recent hypothesis relies on the dysregulation of the epithelial barrier, which might result in the allergen uptake as a primary defect in the pathogenesis of allergic reactions. There is a wealth of literature on T cell responses in allergic diseases [7,8], and thus the objective of this review is to focus on putative modified epithelial functions related to allergy [9-11].

### Epithelium as an active barrier

Respiratory epithelium is the first barrier to the external environment and thus crucially important in the protection of the internal environment. Normally three independent and complex networks connect epithelial cells to each other; desmosomes, adherens junctions, and tight junctions [12]. The respiratory epithelium senses changes in its local environment such as the allergen exposure and also actively responds to these changes [13,14].

Interleukin (IL)-25, IL-33, and thymic stromal lymphopoietin (TSLP) are cytokines, which have a role in Th2-type allergic responses [15]. Previously the epithelium was considered only as a barrier, but now it is considered as a central player in controlling the immune function release of innate cytokines-promoting Th2 responses and the activation of local dendritic cells [16-18]. The exposure of airways to aeroallergens induces a rapid release of these three cytokines from the epithelial cells into the airway lumen and initiates an allergic immune response [19,20].

Recently, disruption in the epithelial barrier has been included into the primary defects in allergic diseases. Atopic dermatitis (i.e. atopic eczema) is an allergic disease characterized by the defective epidermal barrier function, immunological dysregulation, and IgE-mediated sensitization to pollen allergens and food [21,22]. Loss-of-function mutations in the filaggrin gene are linked to atopic dermatitis and other related diseases including peanut allergy [22]. Filaggrin dysfunction has been suspected to cause the impaired barrier function in the skin allowing the allergen penetration and followed by a local inflammation. To more directly verify the link between mutated filaggrin and the barrier dysfunction an elegant survey with wasp allergies was conducted. Wasp stings bypass the skin surface and antigen is injected deep into the dermis [21]. Patients with atopic dermatitis have an increased frequency of the most common filaggrin null mutations (R501X and 2282del4) compared to those with wasp allergy and healthy subjects. This data supports the role of mutated filaggrin in the epithelial dysfunction resulting in the allergic sensitization [21].

The respiratory epithelium is constantly facing hostile injurious stimuli causing damage. Proliferation and differentiation of resident progenitor and stem cells repair the damages and try to maintain the protective barrier [23]. The concept of unified airways suggests that in addition to the anatomic continuity, also inflammation influences the homeostasis of other parts of the airway [24-26]. *In vivo *challenge with the birch pollen allergen, Bet v 1, leads to instant leukocyte extravasation into conjunctival and nasal mucosa only in patients allergic to birch pollen, but not in healthy subjects.

### Allergen transport through the epithelium

Several of the known allergens are proteases [27,28]. Impaired epithelial barrier function can be a direct consequence of proteolytic activity of inhaled allergens, including house dust mite (HDM) [29]. After causing injury to the epithelium Der p 1 m, the major allergen in HDM, can enter the tissue and induce mediate inflammation via Toll-like receptor 4 (TLR4) [30,31]. Der p 2, another allergen from HDM activates respiratory epithelial cells, indicating that this non-proteolytic allergen, in addition to its immunogenic properties, can aggravate a respiratory airway disease by an adjuvant [32].

Also toxic perturbations such as exposure to cigarette smoke can induce epithelial cell gaps and a decrease in trans-epithelial resistance [33]. Cigarette smoke exposure induces also a significant increase in the allergen uptake to epithelium. Thus smoking reduced the barrier function of the respiratory epithelium for allergens and contributed to exacerbation of an allergic disease [33].

A new concept is to focus on the aberrant innate responses of the respiratory epithelium to viral or bacterial infections and how these responses may increase the risk of allergic diseases [34]. As novel sequencing techniques are emerging the role of the resident respiratory microbiome and its influence on the local immune response can now be studied more efficiently [35,36].

The very rapid increase in the incidence of allergic diseases over the last 30 years and the differences in the prevalence of an allergic disease between industrialized and developing countries both suggest that something else than variations in human genetics is the cause of this allergic epidemic [5,6,37]. These observations led to the generation of the "hygiene hypothesis", i. e, the lack of an early microbial stimulation or an exposure to soil and dirt results in dysregulated immune responses to allergens later in life [6,38]. The respiratory epithelium is the home to microbiome, which is estimated to be composed of 10 × 10^12 ^microbes, with a diversity of greater than 1,000 species within each individual [39]. The international Human Microbiome Project aims to define the human microbiome from various locations within the body both in health and in various diseases [40,41]. Several different combinations of local microbes can contribute to the local environment and thus alter the barrier functions of the respiratory epithelium.

Allergen-containing subpollen particles from timothy grass were shown to first bind to and then be internalized by primary epithelial cells in a dose dependent manner. The binding was enhanced with the surfactant protein -D and coincided with the secretion of Interleukin (IL)-8 [42]. Timothy grass pollen allergen Phl p 1 activated respiratory epithelial cells by a non-protease mechanism [43]. Enhancement of TGF-beta, IL-6 and IL-8 induced by Phl p 1 might provide an indirect mechanism through which the allergen may cross the epithelial barrier. Furthermore, recent observations also indicated that timothy grass allergen Phl p 1 is actively transported though the epithelium [44].

**Figure 1:**
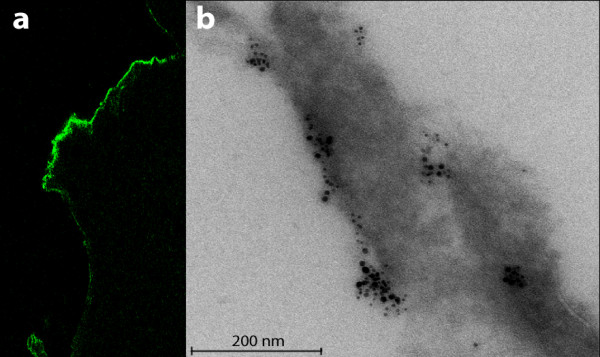
**Bet v 1 binds to and is transported through the conjunctival epithelium only in allergic, but not in healthy subjects**. **Panel a; **A conjunctival epithelial biopsy taken from an allergic patient 1 min after the *in vivo *birch pollen challenge indicated a clear Bet v 1 location detected with anti- Bet v 1 antibodies (green staining for Bet v 1). Original magnification × 200. **Panel b; **The strong clustered staining of anti-Bet v 1 detected with gold label particles at the epithelial villus. Bet v 1 was seen to co-localise with caveolin 2 as shown with double immunoTEM using an anti- Bet v 1 10 nm gold-labelled antibody and anti-caveolin 2 5 nm gold-labelled antibody, magnification × 27 500. Bet v 1 was almost solely (75%) co-localised with a caveolar marker protein caveolin 2.

Already one minute *in vivo *challenge with birch pollen caused Bet v 1 binding to conjunctival epithelial surfaces in the allergic, but not in the healthy individuals (Figure 1a) [45]. Bet v 1 entry into the epithelium was evident also in immuno transmission electron microscopy (TEM) in the allergic, but not in the healthy individuals. One minute after the birch pollen perturbation, before the fulminant clinical symptoms, Bet v 1 was found both on the cell surfaces of villae as well as within the villae, in the cytoplasm, in intracellular vesicles, and also in the nuclei of epithelial cells in allergic patients [45]. At the same time there was no Bet v 1 in the conjunctival epithelial cells of the healthy subjects (Cell level, Table 1). In conjunctival biopsies anti-Bet v 1 staining was seen dispersed into the more basal epithelial cell layers (Tissue level, Table 1). These observations support a very rapid traffic through the epithelium in allergic patients, but not in healthy subjects. Currently the putative receptor(s) mediating this allergen binding and facilitating its transport through the epithelium is not known. A striking specificity is observed when birch pollen allergic subjects were also challenged with timothy grass pollen and no entry of this pollen allergen Phl p 1 into epithelial cells was detected (R. Renkonen unpublished data). While the specific transport mechanism for birch pollen remains unsolved the first hints of the role of caveolae in this have been obtained. In the double immunoTEM stainings caveolin 2, but not caveolin 1 or 3, was present on the conjunctival epithelial surface in the same clusters as Bet v 1 (Figure 1). A great majority of anti-Bet v 1 staining in the epithelial cells was colocalized also with another caveolar marker, flotillin 2. Thus the observed Bet v 1 traffic could involve an active lipid raft and caveolar-dependent transport [46].

**Table 1:** Bet v 1 distribution in the epithelial cells and tissues in allergic patients.

		Distribution of Bet v 1
Cell level	On the surfaces of villi	18%

	Within villi	11%

	Within cytoplasm	51%

	Within vesicles	17%

	Within nuclei	3%

Tissue level	On the apical surface	7%

	Epithelial cell layer 1	29%

	Epithelial cell layer 2	26%

	Epithelial cell layer 3	22%

	Epithelial cell layer 4	16%

Note that the epithelium of healthy patients neither bound nor transported the Bet v 1 allergen.

While the above data suggests a caveolar-dependent traffic, the primary contact with the epithelium is still unknown. Small proteins like cholera toxin or much larger cargo like full *Pseudomonas *bacteria can be transported to and through epithelial cells via lipid raft and caveolar mechanisms [47]. In the gastrointestinal tract food proteins are the targets for lysosomal degradation while selective food allergens are protected from this when they are transported across the intestinal epithelium by their binding to cell surface IgE/CD23 (FcεRII) complexes. The presence of IgE/CD23 on the intestinal epithelium provides means for intact transcytosis for food allergens across the epithelium, and this can lead to classical mast cell-dependent type I anaphylactic allergic reactions [48]. So far this direct interaction with IgE and its low affinity receptor CD23 has been detected only in the intestinal but not in the respiratory epithelium. However, as the transport of pollen allergens through the respiratory epithelium seems to be specific, e.g., our patients allergic to birch pollen do not transport timothy grass allergen and *vice versa*, it is most likely antibody-dependent. Some other allergens are actively and specifically transported through the respiratory epithelium. Ovalbumin allergen (OVA) is taken up rapidly via the respiratory epithelium both in the nose and lower airways in a rodent model [49]. Also, human respiratory epithelial cells internalize timothy grass pollen allergen, Phl p 1 [44].

### Bet v 1 associated epithelial caveolar proteins

Microscopy provided evidence for the novel hypothesis of active allergen transport. This led us to clarify whether the Bet v 1-associated epithelial proteins act as putative receptors in this intraepithelial traffic. With the help the covalently matrix-bound Bet v 1 antigen and nasal epithelial protein lysates collected from both healthy and allergic subjects we began to identify these interacting proteins. By extensive analyses applying both database-related and *de novo *identifications we generated a shortlist of Bet v 1-binding epithelial proteins found only in the allergic patients. Six out of these sixteen proteins (40%) had previously been identified as caveolar proteins: ACTG, ANXA2, CALM, KCNA5, PLEC1, STML2 (Table 2, [46]). While the entire human proteome consists of less than 1% of caveolar proteins, the Bet v 1 associated list of proteins was highly enriched with the caveolar proteins. This observation provided independent support for the observation that Bet v 1 could be transported actively through the epithelium within caveolae at the onset of an allergic reaction.

**Table 2:** Bet v 1-associated nasal epithelial proteins found in the allergic, but not in the healthy subjects.

Proteins found in caveolae and lipid rafts.	
**Name**	**Functions**

ACTG, gamma actin	A subunit of microfilaments, one of the three major components of the cytoskeleton

ANXA2, Annexin 2	Belongs calcium-binding proteins, suppresses phospholipase A2 and thus inhibits inflammation.

CALM, Calmodulin	Belongs calcium-binding proteins, regulation of nuclear transport.

KCNA5	Voltage-dependent potassium ion permeability, has a role in regulating the secretion of insulin

PLEC1, Plectin	Intermediate filament binding protein

STML2, Stomatin 2	Colocalized at intercellular junctions and regulated gap junctions and lipid domain organization.

	

Other epithelial proteins	

**Name**	**Functions**

CROCC, Ciliary rootlet	A cytoskeletal-like structure in ciliated cells

DCD, Dermcidin	Displays antimicrobial activity thus limiting infection by potential pathogens, such as *E.coli *and *S.aureus*.

ECHB	Participates in lipid metabolism.

EPIPL, Epiplakin	A cytoskeletal linker protein, interactions between epiplakin and intermediate filaments

GNDS, Ral/RalBP1	Plays a role in endocytosis and vesicle sorting and migration.

K1C18, Cytokeratin 18	A subunit of intermediate filament, one of the three major components of the cytoskeleton

MARCO	Scavenger receptor, a major mediator of non-opsonized *E. coli *phagocytosis clearance of bacteria *in vivo*

MYCB2	Mediates ubiquitination and subsequent proteasomal degradation of target proteins

S100P	Overexpressed in epithelium in psoriasis, wound healing, skin cancer, inflammation, calcium-binding

TBA8, Alpha-tubulin	The major constituent of microtubules, one of the three major components of the cytoskeleton.

### Bet v 1 associated epithelial caveolar lipids

The primary contact between the allergen and the epithelium can be mediated by lipids. Bet v 1 has been shown to bind cholesterol and enter the epithelium of allergic patients in cholesterol- and glycolipid-rich caveolae [50-52]. We performed 3-D modeling of Bet v 1 and its possible lipid ligands. We modeled a large group of putative lipid ligands for Bet v 1 on the atomic level with a computational ligand docking using the CDOCKER algorithm, which is based on the CHARMm force field engine [53].

We conducted 3-D docking with both experimentally verified Bet v 1 ligands as well as with larger lipid molecules for which experimental affinity studies were not available. *In silico *data suggested that Bet v 1 can bind also to more complex amphiphilic molecules like ceramides, sphingomyelins and even glycolipids, all of which are present on lipid rafts and caveolar surfaces [53]. Thus a binding model was drafted, where the hydrophobic tail groups of lipids are located in the central Y-shaped cavity of the Bet v 1 molecule, while the polar head group points out from the molecule. Taken together, these data provide a hypothesis on how Bet v 1 could directly interact with epithelial surface lipids [53]. It remains to be verified experimentally whether any of these lipids actually participate in the birch pollen uptake on the epithelial surfaces during the early phases of allergic reactions.

### Allergen induced responses on the epithelium

A hypothesis-free approach in order to analyze the differences between respiratory epithelial swabs from allergic and healthy subjects during the asymptomatic winter period [45,46,54], 182 significantly differentially expressed transcripts (and thus proteins after converting them to corresponding proteins) in between these two groups could be detected. Twenty-two epithelial cell surface receptors displayed enhanced expression levels in allergic compared to healthy subjects and eight out of them were found in lipid rafts/caveolae according to manual PubMed literature search (Table 3, [45]). The kinetics of the Bet v 1 caveolar traffic resembles in many ways other previously known caveolar-dependent phenomena, such as the entry of bacterial toxins, whole viruses, bacteria or even parasites [55-61].

**Table 3:** Membrane-bound receptors associated with lipid raft and/or caveolae and displaying upregulated transcript expression levels in allergic compared to healthy conjunctival specimens.

**ARBK1**, Beta-adrenergic receptor kinase 1	Specifically phosphorylates the agonist-occupied form of the beta-adrenergic and closely related receptors.
**CD59**, CD59 glycoprotein	Potent inhibitor of the complement membrane attack complex (MAC) action. Involved in signal transduction for T-cell activation complexed to a protein tyrosine kinase.

**EFNB3**, Ephrin-B3	May play a pivotal role in forebrain function. Binds to, and induces the collapse of, commissural axons/growth cones *in vitro*.

**EPHA1**, Ephrin type-A receptor 1	Receptor for members of the ephrin-A family. Binds with a low affinity to ephrin-A1

**P2RY4**, P2Y purinoceptor 4	Receptor for UTP and UDP coupled to G-proteins that activate a phosphatidylinositol-calcium second messenger system.

**SSR4**, Somatostatin receptor type 4	The activity of this receptor is mediated by G proteins, which inhibit adenylyl cyclase.

**TNFL8**, Tumor necrosis factor ligand superfamily member 8	Cytokine that binds to TNFRSF8/CD30. Induces proliferation of T-cells.

**TRPA1**, Transient receptor potential cation channel subfamily A member 1	Has a central role in the pain response to endogenous inflammatory mediators and to a diverse array of volatile irritants, such as mustard oil, garlic and capsaicin.

A short characterization from SwissProt is included.

In another approach the response of epithelium to the birch pollen challenge was analyzed. Nasal epithelial cell swabs were collected from both allergic and healthy subjects in winter (no symptoms) and during the birch pollen season in May when allergic patients suffer from clinical symptoms. The allergic subjects displayed 331 transcripts between these two time points [46]. Surprisingly the healthy subjects displayed a much stronger response, while they remained without any clinical symptoms through the experiment. Out of the 605 transcripts recorded as altered in healthy controls, the Gene Ontology (GO) category Immune response was the most significantly upregulated. This immune response was not present in allergic subjects giving us the first hint to that allergy might be, at least partially, caused by lack of protective immune defense at the epithelial surfaces [46]. These results suggest that to truly learn about the pathogenesis of diseases one must analyze also how the healthy subjects respond.

A precise time-series analysis was performed with allergic patients and healthy control subjects. The first nasal epithelial specimens were obtained in February when all subjects were symptom-free and the following four nasal epithelial cell swabs were collected from each subject at weekly intervals starting in mid-April when the birch pollen season in Finland begins. While the healthy subjects remained totally symptomless throughout this whole period of time the allergic subjects began to have symptoms already in April, which worsened along with the birch pollen season [54].

Patients allergic to birch pollen showed 105 uniquely regulated nasal epithelial transcripts (converted to proteins for further analyses) with increasing clinical symptoms. Dyneins were among the most enriched Gene Ontology (GO) categories in the list of proteins. Dyneins are molecular motors for the epithelial intracellular caveolar cargo traffic along the microtubuli and ciliary movements [54].

The epithelial cells collected from healthy subjects displayed 22 unique and significantly altered transcripts during the birch pollen season when compared to the winter season. Forty percent of these uniquely regulated genes, i.e., CXCL6, CXCL9, CXCR2, CXL10, FPR1, IL1 and IL8, NKG7, and PGSG belonged to the Gene Ontology category Immune response (GO:0006955). This Immune response category contains altogether only few percent of all annotated proteins of the human proteome (amigo.geneontology.org). Thus the birch pollen allergen induced a strong epithelial immune response in healthy subjects, while this response was lacking from the epithelium of allergic patients [54]. It is remarkable that a similar immunoresponse has been documented on primary human nasal epithelial cells isolated form healthy subjects after HMD challenge and that this response is lacking from the epithelium of allergic subjects. Here also the number of modified transcripts was significantly larger in healthy epithelium that in the allergic (555 compared to 301 probe sets). Many transcripts remain unaffected by the HDM-exposure in the allergic epithelium while these same transcripts were modified by HDM in the healthy epithelium. Genes involved in immunoresponse and with this expression pattern are: IL-1b, IL-8, CXCL2, CXCL3, CCL20, CTGF, HBEGF, and AREG [14,62,63].

How does the epithelium detect danger and respond to it? There are extensive recent reviews on the topic [14,64]. In short the respiratory epithelium continuously senses the changes in its environment. There are several families of receptors to carry out this including eleven human Toll-like receptors (TLR1- 11). They are all membrane bound receptors which mediate effects through dimerization and complex downstream signaling [65]. Other receptors involved in the initiation of epithelial innate immunity include the families protease-activated receptors (PAR1-4), NOD and leucine-rich repeats containing receptors (NLRs) including NOD1 and NOD2 [14,64]. Although a great number of research data has been published on this field with a special reference to the anti microbial defense the true role of this arm on immunology in the development of allergies and asthma remains to be solved [66,67].

## Conclusions

Could allergy be either a epithelial disease or a disease of the immune system? So far it seems that both answers are correct. There is a wealth of immunological studies indicating an altered regulation of IgE production, Th2-response, and other hyperimmune responses, which we do not discuss further in this Review. The role of the epithelial barrier function in allergies has recently also been identified. Filaggrin mutations have been identified constantly in atopic dermatitis and this provoked increasing interest in studies trying to identify the epithelial barrier dysfunction in allergic diseases [68,69]. It is also interesting that mutations in the filaggrin gene not only cause atopic dermatitis, but also lead to a significantly increased risk for other allergic diseases and asthma in the context of eczema [70]. The defective airway epithelial barrier functions together with disrupted tight junctions and innate immunity are also encountered in asthma patients. This could explain the significant impact of viruses and bacteria in acute asthma exacerbations [71,72]. The expensive genome-wide association (GWA) studies have yielded several hypothesis-free candidate genes with known or suspected links to epithelial barrier functions for further analyses in allergic diseases [73,74]. Finally novel approaches pursuing to measure epithelial proteomics [75] and lipidomics [76] will become possible in near future thus allowing us to broaden the DNA and mRNA based GWA analyses.

Taken together, new data has been generated on the role of modified epithelial barrier functions in the early phases of allergic diseases. Active transport of allergens through the epithelium might be incorporated to the pathogenesis of allergy. It is possible that the healthy epithelium displays a strong immune response against pollen allergens and thus escapes from becoming allergic. This challenges the concept of hypersensitivity as the only driving force behind allergic reactions. In fact, if allergy turns out to be, at least in part, a result of epithelial hyposensitivity, it could have major consequences in the strategies of prevention and treatment of these diseases. Towards this end, a national allergy program was launched in Finland in 2008, which changes the basic idea of trying to avoid allergens to the concept of natural exposure and tolerance [6].

## Competing interests

The authors declare that they have no competing interests.

## Authors' contributions

All authors drafted and approved the final manuscript.
